# Growth performance, carcass composition, physico-chemical traits and amino acid profile of meat depending on wormwood (*Artemisia absinthium* L.) dietary supplementation in broilers

**DOI:** 10.5194/aab-67-1-2024

**Published:** 2024-01-05

**Authors:** David Zapletal, Radka Dobšíková, Vlastimil Šimek, Josef Kameník, František Ježek

**Affiliations:** 1 Department of Animal Breeding, Animal Nutrition and Biochemistry, Faculty of Veterinary Hygiene and Ecology, University of Veterinary Sciences Brno, Brno, 612 42, Czech Republic; 2 Department of Animal Origin Food and Gastronomy Sciences, Faculty of Veterinary Hygiene and Ecology, University of Veterinary Sciences Brno, Brno, 612 42, Czech Republic

## Abstract

The present study was conducted to determine the effect of dietary inclusion of the wormwood (*Artemisia absinthium* L.) meal on growth performance, carcass composition, physico-chemical traits and amino acid profile in meats of fattened broilers. In a completely randomised block design, a total of 288 female broilers that were 21 d old (Ross 308) were divided into four dietary groups and fed for 3 weeks: the control basal broiler diet (C), without any anticoccidial or wormwood herb (WH) supplementation, and the C diet plus 10 g (WW1 group), 50 g (WW5 group) or 100 g (WW10 group) of WH meal supplementation per kilogram of basal diet. At the end of the experiment (day 42), broilers were randomly selected for carcass composition and meat quality trait evaluation. In conclusion, the final live weight of chickens was not affected by diets with higher WH levels (*P>*0.05). For the entire experimental period, the feed conversion ratio raised with an increasing WH level in diets, showing the highest value in chickens of the WW10 group (*P<*0.01). Dietary supplementation with wormwood (WW) had no negative effects on the carcass composition or on the chemical and physical traits of meat quality assessed. By contrast, it can be assumed that WH dietary supplementation influenced, predominantly, proteosynthesis of chickens, resulting in alteration of amino acid profiles in meats, where especially increasing aspartic acid and valine contents (*P<*0.001) in the leg meat were found. Our findings indicate that the supplementation of 5 % WH to the diet showed favourable results for chicken performance. However, it is necessary to conduct further studies dealing with WH dietary effects on metabolism and heath control in chickens.

## 1 Introduction

Poultry meat is expected to have the highest demand in the meat industry by 2050. By providing high-quality protein and essential nutrients at a relatively low price, poultry meat plays a key role in improving nutrition in countries worldwide (Mottet and Tempio, 2017). Considering rapid growth of the broiler farming industry and the fact that broilers continuously suffer from stress burden, it is extremely important to supplement the diet with modulators which are able to mitigate the adverse effects of stressors (Shi et al., 2022).

In the poultry industry, feed often contains medications used therapeutically to improve the health, well-being and production performance of birds. The ban on antibiotic growth-promoting compounds in poultry diets in Europe since 2006 has led to the search for natural alternatives, improving performance, protecting animal health and providing profitable production. Regarding the safety of the origin of meat, the supplementation of animal feed with natural phytogenic additives appears to be very promising (Puvača et al., 2014; Csernus et al., 2023). Probiotics, prebiotics, organic acids and herbs, as well as herbal essential oils or aromatic plants and their extracts, have been added in animal feed as alternatives to synthetic growth promoters (Kostadinović and Lević, 2012; Amin et al., 2022; Hassan et al., 2022; Gumus and Gelen, 2023).

*Artemisia absinthium* L., known as wormwood herb (WH), belongs to the plant family Asteraceae and is used as a stomachic, antiseptic, antispasmotic, carminative, cholagogue, febrifuge and anthelmintic substance. The extracts of *Artemisia* spp. exhibit antimicrobial and antioxidative activity; they have also proven to have cytotoxic, neuroprotective, antihepatotoxic, antioxidant, anti-inflammatory, antimalarial, antitumor and antidiabetic features (Fiamegos et al., 2011; Cetin et al., 2019; Sharifi-Rad et al., 2022). The numerous phenols and flavonoids (quercetin, apigenin, flavone, kaempferol, catechin, myristin, artemetin, etc.), as well as vitamins, minerals, and amino acids in *Artemisia *spp., make WH a potential botanical feed additive for animals (Wan et al., 2018; Amin et al., 2022). Wormwood also contains some terpenoids (e.g. 4-terpineol, camphene, chamazulene, *γ*-terpinene), cumarine, caffeic acid, essential oils, (such as camphor, E-caryophyllene, eucalyptol, germacrene D and *α*-cadinol), a sesquiterpene lactone artemisinin, phytosterols (*β*-sitosterol, stigmasterol, campesterol and ergosterol), acetylenes, absinthin and anabsinthine, thujone, tannic, and resinous substances like malic and succinic acid (Tariku et al., 2011; Vieira et al., 2017; Batiha et al., 2020; Amin et al., 2022).

Artemisinin and its derivatives represent the new phytochemical class recommended for the treatment of selected bacterial and parasitic occurrences that are resistant to quinoline drugs. Concerning the antioxidant activity, the above-mentioned compounds show a strong radical scavenging role against hydroxyl ion and hydrogen peroxide. The bactericidal effects of the *Artemisia* spp. compounds are based on involving the destruction of the bacterial membrane on Gram-negative or Gram-positive bacteria (Kshirsagar and Rao, 2021).

Besides that, WH leaves and stem contain bitter compounds, mainly thujone (Lee et al., 2013), which can prevent excessive feed consumption without restriction. In this way, they may be used in the management of feed intake of broilers without any adverse effects (Cetin et al., 2019).

As not many authors published data on WH chemical composition and its effect on health status in terms of its antioxidant, anti-inflammatory, antimicrobial, antiparasitic and other features, limited information is available on its impact on the broiler production efficiency and quality of the chicken meat. The aim of the present study was to evaluate the effect of feed supplemented with different levels of wormwood (*Artemisia absinthium* L.) on growth performance, body and carcass composition, physico-chemical characteristics, and amino acid profile of breast and in leg meat of broilers. In line with the literature sources, the hypothesis of this research is that the diets with WH meal supplementation will result in the broilers' lower growth dynamics and will have no negative effects on the carcass composition or chemical and physical traits of meat quality.

## 2 Material and methods

### 2.1 Material and methods

The experimental procedures were approved by the Animal Welfare Committee of University of Veterinary Sciences Brno (project no. 19-2021). A total of 288 female Ross 308 broilers that were 21 d old were used in the experiment. Broiler chickens were randomly divided into four dietary groups (one control and three experimental; 72 birds per group). Chickens were weighed, and birds from each group were housed in four floor pens (four replicates; 18 birds/replicate) covered with wood shavings. Broilers were housed in the accredited experimental stable of the Department of Animal Breeding, Animal Nutrition and Biochemistry, University of Veterinary Sciences Brno, under controlled housing conditions that fully satisfied the standards used for fattening of Ross 308 broilers (Aviagen, 2018).

### 2.2 Experimental design

The two-phase feeding program (grower and finisher) was used. At first, a 4 d gradual changeover from the starter diet to the experimental grower diets was performed. The grower and finisher diets were offered from day 21 to 35 and day 36 to 42, respectively. The composition of the basal diet is outlined in Table 1. Mastercube^®^ is a low inclusion pellet binder. It is composed of a synergistic blend of polysaccharide gums, starch and mineral hardener. The chickens in the control (C) group were fed basal diets without any anticoccidial products or WH supplementation. For the experimental groups, the powder from whole aerial parts of WH (*Artemisia absinthium*) was added to the basal diets; the chemical composition of WH powder is outlined in Table 2. The experimental WW1, WW5 and WW10 groups were given the diets with the addition of 10, 50 and 100 g WH, respectively, per kilogram of basal diet; the ingredient and nutrient compositions of experimental diets are outlined in Table 3. The experimental period lasted for 21 d. Feed and water were supplied ad libitum. Broilers were observed for any signs of illness and behavioural changes twice a day. On days 21, 28, 35 and 42, the broiler chickens were weighed, and feed intake (FI) on a per pen basis was recorded. Mortality was recorded daily. The average live weight (LW) gain and FI adjusted for mortality were used to calculate the feed conversion ratio (FCR; feed / gain).

**Table 1:**
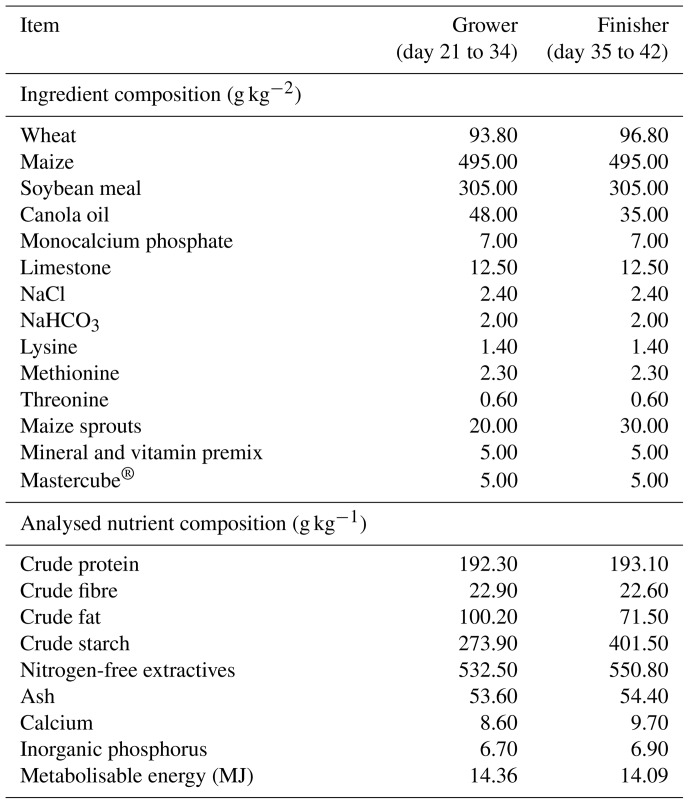
**Table 1**Ingredient and chemical compositions of the basal diets as fed basis.

**Table 2:**
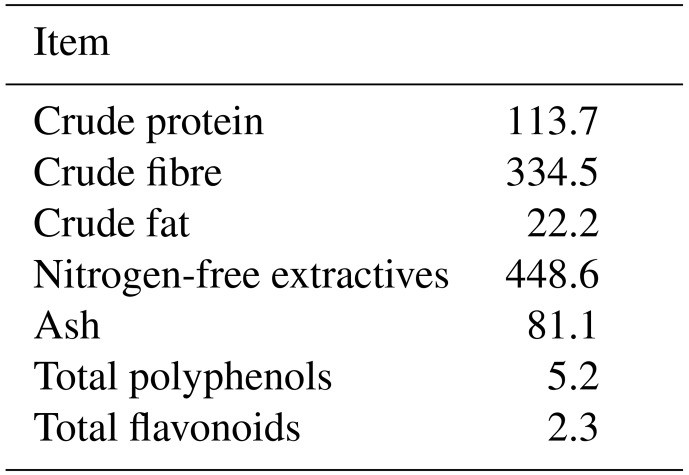
**Table 2**Chemical composition of wormwood herb on dry matter basis (g kg^−1^).

**Table 3:**
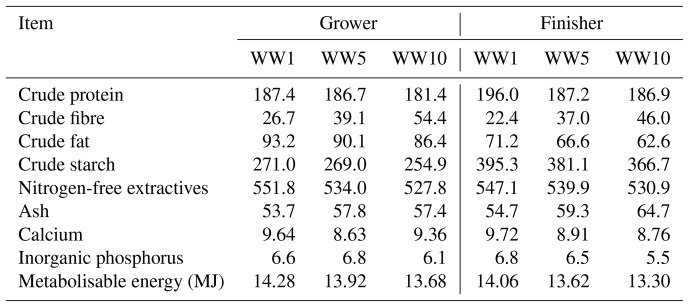
**Table 3**Chemical composition of experimental diets as fed basis (g kg^−1^).

### 2.3 Slaughter, body dissection and meat samples

At the end of the experiment (day 42), 16 broilers (4 birds per replicate) per dietary group were randomly selected for carcass evaluation. Birds were weighed prior to slaughter; thereafter, they were stunned and exsanguinated by cutting the jugular vein. Dissection of the chicken body and carcass processing were performed according to procedures described by Zapletal et al. (2017). Briefly, feathers were removed after scalding. Each bird was processed by removing the head, shanks and feet. Evisceration was performed by removing the viscera without kidneys and disrupting the fat pad. The neck with skin was removed. The carcass, neck with skin, heart, liver, gizzard, both legs (thighs and drumsticks), leg meat, breasts with skin, and breast muscles (minor and major) were weighed. The yield of particular body components was calculated as their intrinsic weight to the slaughter weight (SW) of broilers. Thereafter, right leg and right breast with skin were sampled and delivered to the laboratory for assessment of physical characteristics of chicken meat. Finally, samples of leg and breast meat were packed and stored at – 20 ^∘^C until the analysis for selected chemical characteristics was performed.

### 2.4 Laboratory methods

In feeds and WH powder, the following nutrients were determined: crude protein (CP), crude fibre, crude fat (CFa), crude starch (CSt), nitrogen-free extractives, crude ash, calcium (Ca), inorganic phosphate (P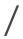 1000). As for WH powder, contents of total polyphenols and total flavonoids were also determined. For this purpose, 100 mg of the dried tissue was extracted into 1 mL of methanol using 10 min of sonication and 3 h of incubation (1400 rpm, 50 ^∘^C). The resulting extract was diluted 10 times into methanol. Total polyphenols were determined using the Folin–Ciocalteu method, and its content is expressed as gallic acid equivalents. Total flavonoids were determined according to the method published by Chandra et al. (2014), and its content is expressed as quercetin equivalents.

The analyses used for determination of proximate chemical composition and amino acid (AA) content in leg and breast chicken meat were performed according to laboratory methods described by Gál et al. (2022).

The measurement of pH values was conducted using a measuring system comprised of a combined needle-tipped electrode – TipTrode combination pH puncture electrode (Hamilton, USA). Electrodes of this type contain an EVEREF reference electrode that minimises the influence of the sample temperature on the potential of the reference system. The electrode is designed for measurements of tougher samples and samples with a lower water content in the food industry. The electrode potential was measured with a Thermo Scientific Orion 4-Star mV/pH meter (Thermo Fisher, USA). The system was calibrated by three-point calibration within a range of 4–10 pH. The slope value of the measuring system lay within a range of 93.5 % to 95.2 % of the theoretical slope during the entire measurement period.

The instrumental measurement of the texture (Warner–Bratzler shear force test; W–B) of the cooked meat was performed the day after cooking. The chilled meat samples were tempered to room temperature before measurement. The W–B tests of cooked meat were conducted using the universal test machine (Instron 5544, software IX series, USA). Samples of a rectangular cross section of 1×1 cm (and 2 cm along the fibre axis) were cut from the cooked meat. These samples were sheared at right angles to the fibre axis using a W–B shear blade with a rectangular hole. The blade travelled at 80 mm min^−1^ relative to the sample. The parameter measured from the force deformation curve was the maximum shear force (N). The average of five shear values represented the W–B shear parameter for each analysed sample.

Colour of the meat samples was measured by the International Commission on Illumination (CIE) 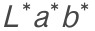 system using a Konica Minolta CM-5 spectrophotometer (Konica Minolta, Japan). The measuring area of 8 mm, illuminant D65 and 10^∘^ standard observer were used. The instrument was standardised to white and black before measurement. CIE L^*^ (lightness), a^*^ (redness), and b^*^ (yellowness) were measured. Five independent subsamples were measured for each sample.

The drip loss test according to Honikel (1998) was used for the determination of water holding capacity. Briefly, according to this method, meat samples were placed in a container on a support mesh and sealed with a lid (note: preferably a plastic cup with a lid of a suitable size so that the meat sample does not touch the walls of the cup). After a period of storage (usually 24 h) at refrigerated temperatures (1 to 5 ^∘^C), samples are reweighed. In the case of this study, a plastic cup with a volume of 500 mL was used; the test lasted 24 h at a temperature of 4 ^∘^C.

### 2.5 Statistic analysis

The arithmetic mean and standard deviation (SD) were determined for LW, average daily gain (ADG), FI, FCR, body composition, and physical and chemical characteristics of meat in respective dietary groups. To test normality of data distribution in above-stated variables within evaluated groups, the Shapiro–Wilk test was used. The normality was proved in all of these variables. Statistical evaluation of data basically followed procedures used by Tůmová et al. (2022a, b). Data of LW, ADG, FI, FCR, body composition and meat characteristics were analysed by a general linear model (GLM) procedure, where the diet was included as a fixed effect and the pen was included as a random effect. The pen was used as an experimental unit for evaluation of growth performance, while the individual broiler represented the experimental unit for evaluation of body composition and meat characteristics. Differences among groups were tested by the honest significant difference (HSD) post hoc test for growth performance and by the Tukey's post hoc test for body composition and meat quality traits assessed. A chi-square test with Yates' correction was used to compare differences in mortality of fattened broilers among dietary groups. The significance was considered at the *P*<0.05 level. All statistical procedures were performed by the STATISTICA CZ, version 10, software (StatSoft Inc., 2011).

## 3 Results

### 3.1 Results

In the course of the experiment, significant differences in LW and ADG of broilers among respective dietary groups were found (Table 4). On day 28, we found a significant decrease (*P*<0.01) in LW of chickens that were fed the diet supplemented with 10 % WH compared to those in the C group. On days 35 and 42, a significant decrease in LW was found in the group WW1 compared to the C group (*P*<0.05). As for ADG, feeding broiler diets supplemented with WH caused significant changes in growth intensity within all three periods assessed (*P*<0.01). In terms of the whole course of the experiment, only the WW1 group showed a lower growth intensity when compared to the C group (*P*<0.05).

**Table 4:**
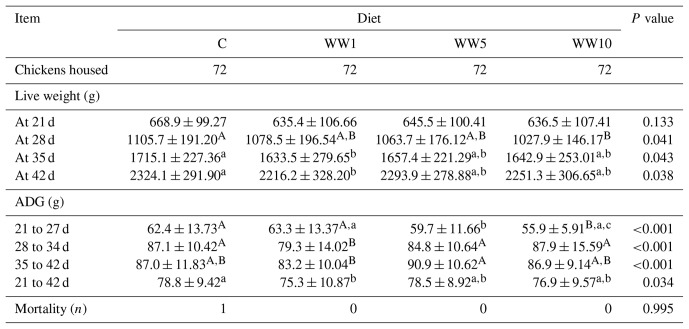
**Table 4**Live weight and ADG of broilers depending on diet in particular periods of fattening (means ± SD).

ADG: average daily gain. 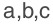: Means within a row with different superscript letters differ (*P<*0.05); ^A,B^: Means within a row with different superscript letters differ (*P<*0.01).

### 3.2 Feed intake and feed conversion ratio

Data on FI and FCR of broilers are given in Table 5. As for FI values, no significant changes (*P>*0.05) among respective dietary groups were found in the course of the experiment. Values for FCR, throughout three particular periods, did not differ among dietary groups. However, regarding the whole course of the experiment, a higher FCR value was found in chickens fed the diet supplemented with 10 % WH when compared to those in the C, WW1 and WW5 groups (*P*=0.002).

**Table 5:**
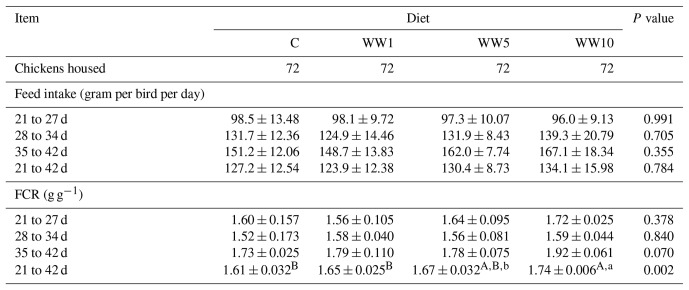
**Table 5**Feed intake and FCR of broilers depending on diet in particular periods of fattening (means ± SD).

FCR: feed conversion ratio. ^a,b^: Means within a row with different superscript letters differ (*P<*0.05); ^A,B^: Means within a row with different superscript letters differ (*P<*0.01).

### 3.3 Body and carcass characteristics

Concerning body and carcass composition of female broilers, the results are given in Table 6. In all particular traits monitored, no significant differences (*P>*0.05) were found among respective dietary groups of chickens slaughtered at 42 d of age.

**Table 6:**
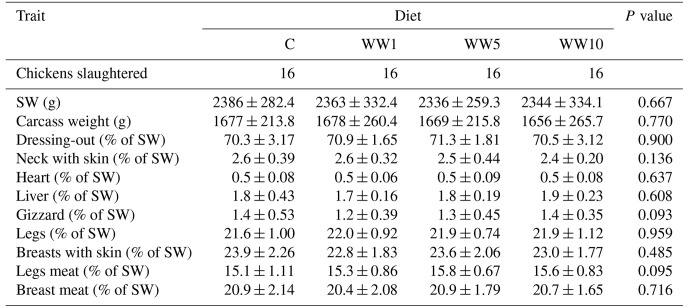
**Table 6**Body and carcass characteristics of 42 d old female broilers depending on diet (means ± SD).

SW: slaughter weight.

### 3.4 Physical characteristics of broiler meat

Characteristics of the physical examination of the female broiler leg and breast meat are presented in Table 7. The addition of the WH powder into the diets had no significant effects (*P>*0.05) on the measured physical characteristics either in leg and breast meat.

**Table 7:**
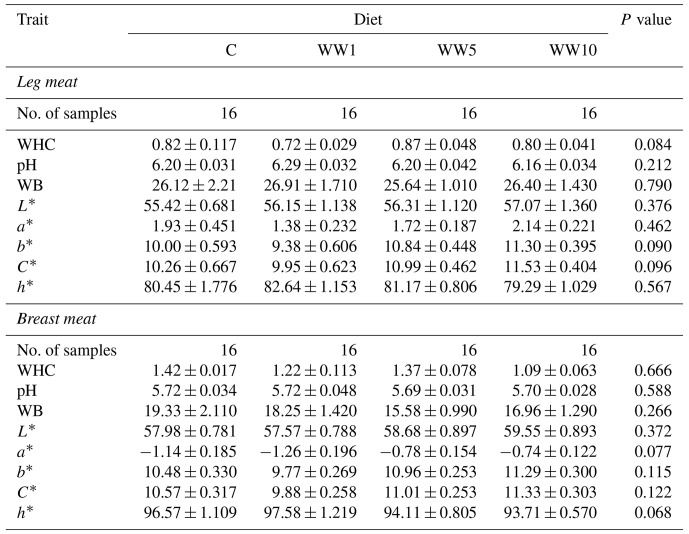
**Table 7**Physical characteristics of female broiler meats depending on diet (means ± SD).

WHC: water holding capacity; WB: Warner–Bratzler shear force; L^*^: lightness; a^*^: redness; b^*^: yellowness; C^*^: chroma; h^*^: hue angle.

### 3.5 Proximal chemical composition

Compared to the C group, only dietary supplementation of 1 % WH resulted in the higher CP content in leg meat and higher dry matter (DM) value in breast meat of broilers (*P*<0.05). All other monitored traits of proximate chemical composition in leg and breast meat were not influenced by respective levels of WH supplementation (Table 8).

**Table 8:**
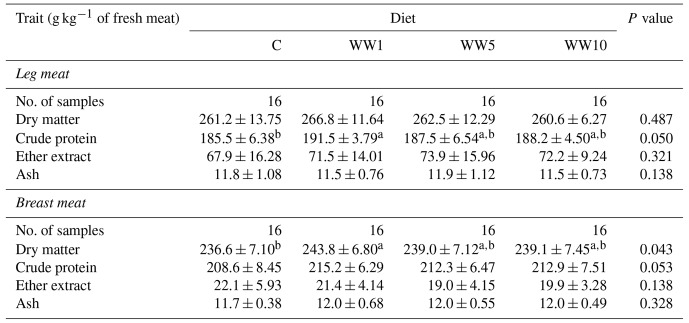
**Table 8**Chemical composition of female broiler meats depending on diet (means ± SD).

^a,b^: Means within a row with different superscript letters differ (*P*<0.05).

### 3.6 Amino acid profile

As for the leg meat (Table 9), dietary WH supplementation affected primarily the contents of Thr, Val, His, Asp, Gly and Tyr (*P*<0.01) as well as the levels of Lys, Ile and Phe (*P*<0.05). Higher levels of WH dietary supplementation (groups WW5 and WW10) led to the increased level of Val and Asp (*P*<0.01) and to decreased levels of Phe (*P*<0.05) and Tyr (*P*<0.01) compared to the C group.

**Table 9:**
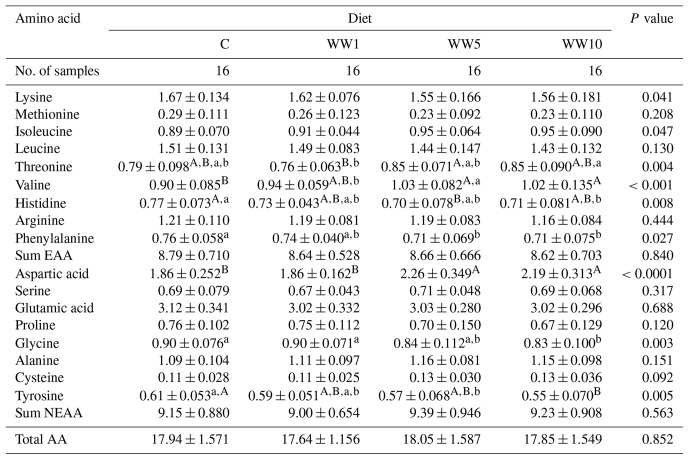
**Table 9**Amino acid profile (g per 100 g of fresh meat) in leg meat of female broilers depending on diet (means ± SD).

^a,b^: Means within a row with different superscript letters differ at *P<*0.05; ^A,B^: Means within a row with different superscript letters differ at *P<*0.01; EAA: essential amino acids; NEAA: non-essential amino acids; AA: amino acids.

Regarding the breast meat (Table 10), dietary WH supplementation mainly influenced the contents of Arg and Cys (*P*<0.01) as well as the levels of Lys, Leu, Thr, His, Phe, Ser, Glu, Gly and Tyr (*P*<0.05). Compared to the C group, the WW1 group displayed lower content of Thr (*P*<0.01) and higher content of Arg (*P*<0.01), the WW5 group showed also higher content of Arg (*P*<0.05).

**Table 10:**
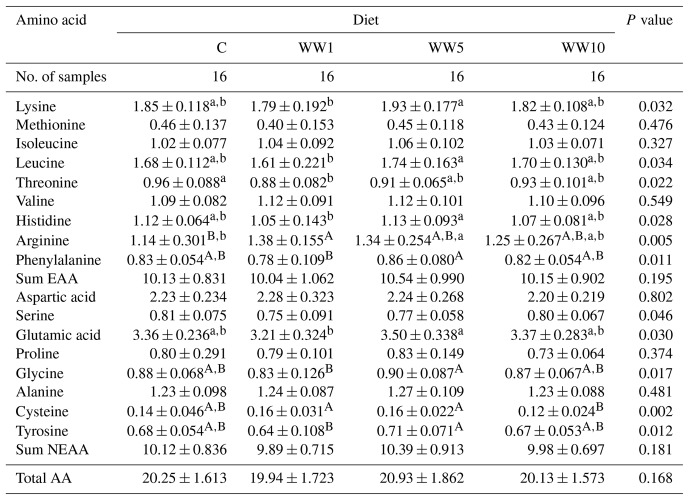
**Table 10**Amino acid profile (g per 100 g of fresh meat) in breast meat of female broilers depending on diet (means ± SD).

^a,b^: Means within a row with different superscript letters differ at *P<*0.05; ^A,B^: Means within a row with different superscript letters differ at *P<*0.01; EAA: essential amino acids; NEAA: non-essential amino acids; AA: amino acids.

## 4 Discussion

### 4.1 Discussion

In the present study, feeding broiler diets supplemented with WH caused significant differences in LW and ADG among dietary groups in monitored periods of the experiment. Within the first week of the experiment, chickens fed diets with higher WH levels (groups WW5 and WW10) showed decreased growth intensity, while in the next 2 weeks (28 to 42 d of age) they increased ADG values that were not different to those in the C group. Moreover, in the last week of the experiment, chickens fed the diet supplemented with 5 % WH displayed a considerably higher growth rate than those fed the diet supplemented with 1 % WH. When compared to the C group, the growth intensity of chickens in the WW5 and WW10 groups did not differ for the entire period of the experiment. Feeding broilers a diet supplemented with 1 % WH led to the lower final live weight and lower growth intensity (in the period between 28 and 42 d of age) compared to those fed C diet. In the study of Cetin et al. (2019), LW of chickens was not affected by feeding diets supplemented with WH in the amount of 1.2 % to 4.7 % within 42 d of fattening, which is not in agreement with findings of the present study. Moreover, WH used in the study of Cetin et al. (2019) contained considerably lower values for CP (6 %) and ash (4.7 %) as compared to WH used in the present study; the levels of CFa and crude fibre were similar. Amin et al. (2022) reported that dried WH powder contains 89 % of dry matter, 18 % of CP, 5 % of ether extract, 18 % of crude fibre, 41 % of nitrogen-free extract, 7.5 % of total ash, 1.1 % of Ca and 0.8 % of P_i_. Levels of respective nutrients in WH are affected by factors such as harvesting time of herb, geographical area and altitude of planting (soil type, etc.), intrinsic nutrient compositions, and contents of bioactive compounds of WH may influence physiology of fed animals. Besides, Kostadinovič et al. (2015) found increased LW in 42 d old Arbor Acres broilers fed diets supplemented with 15 % and 20 % WH compared to those fed a basal diet (*P*<0.01), whereas broilers fed the diet supplemented with 10 % WH did not show different LW than those fed a basal diet. In the study of Kostadinovič et al. (2015) diets supplemented with WH meal were feeding broilers for the entire fattening period (from day 1 up to day 42), whereas broilers of the present study were fed WH diets from the age of 21 d. At the same time, the longer feeding period of WH diets and absence of a diet changeover in the study of Kostadinovič et al. (2015) could result in a different metabolism rate in the younger chickens and thus display a different body gain as compared to broilers in the present study.

### 4.2 Feed intake and feed conversion ratio

In the present study, no differences in FI of chickens were found among respective dietary groups, neither when assessing particular periods of the experiment nor for the entire experimental period. Cetin et al. (2019) observed that higher levels of WH supplementation in the diet did not affect the FI in chickens. In their study, however, a diet supplemented with 1.2 % WH led to an increased FI compared to chickens fed a basal diet throughout the entire period of fattening, so authors state that 1.2 % WH supplementation in the diet was an ideal dose which the chicks were accustomed to. Kostadinovič et al. (2015) found no differences in FI of chickens fed diets supplemented with 10 %–20 % WH compared to those fed a basal diet throughout the entire period of a 6-week fattening period. In their study, however, supplementation of 20 % WH in the diet led to an increased FI in chickens when compared to those fed a basal diet or diet supplemented with 10 % WH in the period between 22 and 42 d of age. In the present study, unreduced FI is a positive finding considering that WH possess a bitter taste and thus this can be problematic for animals to accept in feed, although a bird's ability to perceive the feed taste is limited owing to the low number of taste buds in the oral cavity.

In the present study, FCR values in three respective experimental periods showed non-significant differences among diets tested. However, when assessing the entire period of the experiment, the FCR value raised with an increasing level of WH in diets, showing the highest value in chickens of the P10 group. This increase in FCR can be expected with regard to an increase in a total crude fibre content of the diet. Similarly to the finding of the present study, Kostadinovič et al. (2015) observed worse feed conversion in chickens fed diets with higher levels of WH (*P>*0.05). By contrast, Cetin et al. (2019) found no effect of tested dietary WH concentrations on the FCR value in fattened broilers.

### 4.3 Body and carcass characteristics

In the present study, dietary supplementation with WH powder had no effects on body and carcass composition of female broilers at 42 d of age. The proportion of carcass parts corresponds to the common values found by Viliene et al. (2022) in Ross 308 hybrid fattened chickens. Cetin et al. (2019) found a significantly higher proportion of liver in 42 d old fattened Ross 308 chickens which received a complete diet with 1.17 % inclusion of the *Artemisia absinthium* powder. Also, in the above-mentioned study conducted on Ross 308 chickens, both males and females were found to have generally higher proportions of liver, heart and gizzard compared to the present study conducted on Ross 308 females.

### 4.4 Physical characteristics of broiler meat

Concerning the found values of the particular physical traits of chicken meat, this can be highlighted that dietary WH supplementation had no negative effects on any of all measured traits, although high levels of WH were included in diets. In the present study, however, slightly higher values for lightness and yellowness (*P*>0.05) were observed in leg and breast meats in chickens fed diets supplemented with 5 % and 10 % WH. A similar tendency was also reported by Chu and Park (2022) in pork meat, when pigs were fed diets with WH supplementation at the level of 0.5 % to 1.5 %. Besides, average values for physical traits of meat assessed in this study are in agreement with those reported recently for meat of conventionally fattened broilers (da Silva, 2017; Özbek et al., 2020).

### 4.5 Proximal chemical composition

Regarding proximate chemical composition of meats in the present study, dietary inclusion of WH resulted only in higher levels of CP in leg meat and DM in breast meat in chickens of the WW1 group compared to those in the C group. Kostadinovič et al. (2015) found the reduced level of intramuscular fat in breast meat of 42 d old Arbor Acres chickens fed a diet supplemented with 20 % WH compared to chickens fed a basal diet, which is not in agreement with the results of the present study. In addition, Kostadinovič et al. (2015) observed a tendency toward a higher value of CP in breast meat of chickens fed a WH diet, which is in agreement with the finding of the present study (*P*=0.053). Besides, values of CP and ash content in breast meat of the present study are similar to those found by Valenta et al. (2022) and Tůmová et al. (2022b) in Ross 308 chickens. The fat content in breast meat of chickens in the present study is similar to values observed by Kostadinovič et al. (2015) and by Stęczny and Kokoszynski (2019) in 42 d old Ross 308 females.

### 4.6 Amino acid profile

Total contents of all AA, essential AA (EAA) and non-essential AA (NEAA) profiles in both leg and breast meat did not differ among respective dietary groups of chickens in the present study. However, WH dietary inclusion influenced the levels of some AAs within EAA and NEAA groups in both assessed meats. In case of EAAs, a higher value for Val and lower values for His and Phe were found in leg meat of chickens fed higher levels of WH supplementation (groups WW5 and WW10) in diet compared to chickens of C and WW5 groups. In breast meat, the C group displayed the higher value for Thr and lower value for Arg when compared to the WW1 group. Regarding NEAAs, the lower value for Asp and higher values for Gly and Tyr in leg meat were found in chickens of the C group compared to meat of chickens fed with higher dietary WH supplementation (5 % and 10 %). As for breast meat, levels of some NEAAs (Glu, Ala and Tyr) differed mainly between the WW1 and WW5 groups, while the C group did not differ in its levels when compared to all other groups with WH dietary supplementation. In the present study, higher alteration of respective AA levels in breast meat compared to leg meat of broilers points to different protein synthesis in particular chicken muscle due to WH dietary supplementation. It has been proved recently that dietary inclusion of some herbs or their derived products may alter muscle protein synthesis in chickens (Santoso et al., 2018; Greene et al., 2021), which is in agreement with the results of the present study. Moreover, alteration in the AA profile of raw meat can be also related to meat flavour (Liu et al., 2016) or quality of products after meat processing (Leggio et al., 2012). In addition, the levels of AAs in chicken meats of the present study are similar to those observed by Haščík et al. (2020) in 42 d old Ross 308 chickens.

## 5 Conclusions

In conclusion, dietary supplementation of WH resulted in different growth intensities of female broilers within particular periods of the experiment, while the final LW of chickens was not affected by diets with higher WH levels. For the entire experimental period, the FCR raised with an increasing WH level in diets, showing the highest value in chickens of the WW10 group. Dietary supplementation with WH had no negative effects on the carcass composition or chemical and physical traits of meat quality assessed. In contrast, it can be assumed that WH dietary supplementation influenced, predominantly, proteosynthesis of chickens, resulting in alteration of the amino acid profile in meats. The supplementation of 5 % WH in the diet showed favourable results for chicken performance in the present study. However, it is necessary to conduct further studies dealing with WH dietary effects on metabolism and heath control in chickens.

## Data Availability

Data will be made available upon reasonable request.
